# Long-Term Outcomes of Contemporary Durable Left Ventricular Assist Device Support in Younger Patients: A EUROMACS Analysis

**DOI:** 10.1093/ejcts/ezag234

**Published:** 2026-10-07

**Authors:** Guglielmo Gallone, Kevin M Veen, Alexander M Bernhardt, Kadir Caliskan, Emil Najjar, Ivan Netuka, Anna Mara Scandroglio, Antonio Spitaleri, Diego Sangiorgi, Peter Ivak, Arnt Fiane, Feras Khaliel, Mustafa Özbaran, Yuri Pya, Daniel Zimpfer, Gloria Färber, Jan Gummert, Bart Meyns, Paola Quattroni, Felix Schoenrath, Gaetano Maria De Ferrari, Mauro Rinaldi, Evgenij Potapov, Antonio Loforte

**Affiliations:** Cardiovascular and Thoracic Department, “Città della Salute e della Scienza” Hospital, 10126 Turin, Italy; Department of Medical Sciences, University of Turin, 10126 Turin, Italy; Department of Surgery, Thorax Center, Erasmus Medical Center, 3015 GD Rotterdam, Netherlands; Department of Cardiovascular Surgery, University Heart and Vascular Center Hamburg, 20246 Hamburg, Germany; Department of Cardiology, Thorax Center, Cardiovascular Institute, Erasmus MC University Medical Center, 3015 GD Rotterdam, Netherlands; Department of Cardiology, Karolinska University Hospital, 171 76 Stockholm, Sweden; Department of Medicine, Karolinska Institutet, 171 77 Stockholm, Sweden; Institute for Clinical and Experimental Medicine (IKEM), 140 21 Prague, Czech Republic; Cardiac Intensive Care Unit, IRCCS San Raffaele Scientific Institute, 20132 Milan, Italy; Cardiovascular and Thoracic Department, “Città della Salute e della Scienza” Hospital, 10126 Turin, Italy; Department of Surgical Sciences, University of Turin, 10126 Turin, Italy; PhD programme in Bioengineering and Medical-Surgical Sciences, University of Turin, Politecnico di Torino, 10126 Turin, Italy; Cardiovascular Department, Maria Cecilia Hospital, GVM Care & Research, Cotignola, Ravenna 48033, Italy; Institute for Clinical and Experimental Medicine (IKEM), 140 21 Prague, Czech Republic; Department of cardiothoracic surgery, Oslo University Hospital and Faculty of Medicine, 0372 Oslo, Norway; Adult Cardiac Surgery Section, Department of Surgery, Heart Center, King Faisal Specialist Hospital & Research Center, 11211 Riyadh, Kingdom of Saudi Arabia; Department of Cardiovascular Surgery, Ege University Hospital, 35100 Izmir, Turkey; National Research Cardiac Surgery Center, 010000 Astana, Kazakhstan; Department of Cardiac Surgery, Medical University of Vienna, 1090 Vienna, Austria; Department of Thoracic and Cardiovascular Surgery, University Hospital Würzburg, 97080 Würzburg, Germany; Department of Thoracic and Cardiovascular Surgery, Herz- und Diabeteszentrum NRW, 32545 Bad Oeynhausen, Germany; Department of Cardiovascular Sciences, Katholieke Universiteit Leuven, 3000 Leuven, Belgium; EUROMACS, EACTS House, Windsor SL4 1EU, United Kingdom; Department of Cardiothoracic and Vascular Surgery, Deutsches Herzzentrum der Charité (DHZC), DZHK (German Centre for Cardiovascular Research), 13353 Berlin, Germany; Cardiovascular and Thoracic Department, “Città della Salute e della Scienza” Hospital, 10126 Turin, Italy; Department of Medical Sciences, University of Turin, 10126 Turin, Italy; Cardiovascular and Thoracic Department, “Città della Salute e della Scienza” Hospital, 10126 Turin, Italy; Department of Surgical Sciences, University of Turin, 10126 Turin, Italy; Department of Cardiothoracic and Vascular Surgery, Deutsches Herzzentrum der Charité (DHZC), DZHK (German Centre for Cardiovascular Research), 13353 Berlin, Germany; Cardiovascular and Thoracic Department, “Città della Salute e della Scienza” Hospital, 10126 Turin, Italy; Department of Surgical Sciences, University of Turin, 10126 Turin, Italy

**Keywords:** heart failure, heart transplantation, left ventricular assist device, outcome, HeartMate3

## Abstract

**Objectives:**

Superior survival outcomes have been reported for younger patients receiving durable left ventricular assist devices (LVADs) in Interagency Registry for Mechanically Assisted Circulatory Support (INTERMACS). We explored whether these findings extend to contemporary European practice leveraging on EUROMACS (European Registry for Patients with Mechanical Circulatory Support).

**Methods:**

Adult patients undergoing implantation of current-generation magnetically levitated LVADs in EUROMACS were stratified according to age groups. The primary outcome was 5-year on-device survival, censored at heart transplantation (HT) or LVAD explantation. Secondary outcomes included LVAD explant for myocardial recovery and survival free from major adverse events (bleeding, stroke, device-related infection, or device malfunction). Death incidence accounting for HT and support cessation as competing risks was also calculated.

**Results:**

A total of 2850 patients undergoing HeartMate 3 implantation were included (<50 years: 23.5%; 50-59 years: 29.7%; 60-69 years: 38.0%; ≥70 years: 8.8%). Younger patients had fewer comorbidities, a higher prevalence of non-ischaemic cardiomyopathy, and INTERMACS profile ≤3. Younger patients had superior on-device 5-year survival (<50 years: 74.7%; 50-59 years: 65.5%; 60-69 years: 53.9%; ≥70 years: 48.8%; *P* < .001) and LVAD explantation for myocardial recovery (<50 years: 4.3%; 50-59 years: 1.8%; 60-69 years: 2.3%; ≥70 years: 0.4%; *P* = .031). Accounting for competing outcomes, the 5-year cumulative incidence of death was lowest among younger patients (<50 years: 20.8%; 50-59 years: 29.1%; 60-69 years: 40.8%; ≥70 years: 49.5%; *P* < .001), driven by higher HT rates. Survival free from bleeding was significantly higher in younger patients (*P* < .001). In contrast, 5-year freedom from stroke, device-related infection, and device malfunction was similar across age groups.

**Conclusions:**

In contemporary European practice, patients <50 years undergoing LVAD implant had high 5-year survival approaching that reported in cohorts of patients listed for HT. These findings support further investigation of prolonged LVAD support before HT as a potential strategy to maximize lifetime benefit in selected motivated younger candidates.

## Graphical Abstract

**Graphical Abstract:**
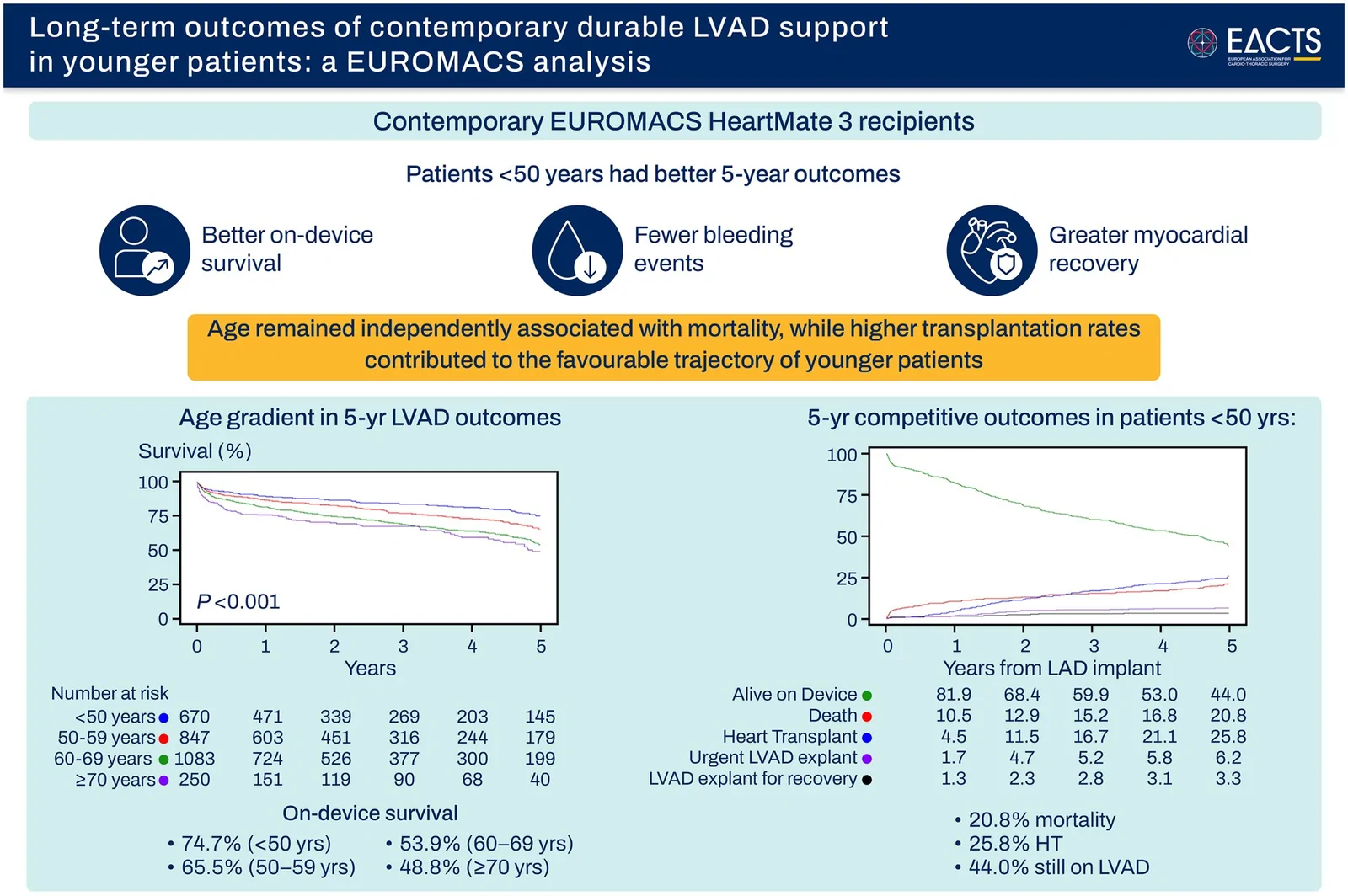

## Introduction

Durable left ventricular assist devices (LVADs) have undergone substantial evolution over the past decade, with marked improvements in survival and reductions in device-related complications. The introduction of fully magnetically levitated centrifugal-flow pumps has been associated with superior long-term outcomes, including lower all-cause mortality, driven by lower rates of pump thrombosis and stroke, and improved overall hemocompatibility compared with prior-generation devices.^1–4^

Against this background, emerging evidence suggests that the prognostic benefit of LVAD support may interact with age. Analyses from the Interagency Registry for Mechanically Assisted Circulatory Support (INTERMACS) demonstrate that younger patients, particularly those <50 years, experience significantly higher survival and lower rates of major adverse events with contemporary magnetically levitated devices compared with older recipients.^5^^,^^6^ In these patients, 5-year survival now approaches that observed after heart transplantation (HT).^5^^,^^6^

These observations have generated interest in the concept of net prolongation of life, in which durable LVAD therapy is used as an initial heart replacement strategy in selected younger transplant-eligible patients, deferring HT to later stages to maximize cumulative lifetime survival.^6^^,^^7^ Such a strategy is particularly relevant given the intrinsic limitations of HT in younger patients: contemporary median post-transplant survival is approximately 12-13 years, retransplantation rates remain low (∼3%), and late post-transplant mortality is frequently driven by complications of chronic immunosuppression—including malignancy, chronic kidney disease, metabolic disorders, and infections—that cannot be mitigated by repeat HT.^8^ Beyond individual outcomes, preferential HT in LVAD-eligible patients may have system-level consequences, as HT in LVAD-eligible patients could increase waitlist mortality among patients with greater acuity or without LVAD options.^9^^,^^10^

Despite the conceptual plausibility of an LVAD-first strategy in younger patients, data on mid- to long-term outcomes during LVAD support remain limited. Importantly, no contemporary European data are available to assess the generalizability of the INTERMACS findings in healthcare systems with distinct allocation policies and patterns of LVAD utilization. Moreover, prior INTERMACS and other analyses have relied predominantly on Kaplan-Meier methods, precluding a detailed assessment of competing outcomes such as HT or device explantation.^5^^,^^6^ Addressing these gaps is essential to inform the role of durable LVAD support within modern heart replacement therapy paradigms. To address these gaps, we leveraged the EUROMACS (European Registry for Patients with Mechanical Circulatory Support) to assess age-specific outcomes and quantify competing risks during contemporary LVAD support in European practice.

## Methods

### Study design and data source

This is an observational, multicentre, registry-based analysis of patients undergoing durable LVAD implantation recorded in the EUROMACS. EUROMACS is a prospective, Europe-wide registry collecting standardized demographic, clinical, procedural, and outcome data from participating centres, according to predefined definitions and quality control procedures.^3^^,^^11^

Adult patients (≥18 years) undergoing primary implantation of a current-generation, fully magnetically levitated LVAD between January 1, 2011, and December 31, 2023, were considered for inclusion. The dataset was locked on June 17, 2024. Only patients receiving a HeartMate 3 (HM3, Abbott) pump were included in this study. Patients receiving a durable biventricular support, total artificial heart implantation, or lacking follow-up data were excluded. The final study population consisted of 2850 patients. Patients were stratified according to prespecified age groups: <50 years, 50-59 years, 60-69 years, and ≥70 years.

### Study outcomes

The primary outcome of the study was 5-year survival on device support, censoring at HT or LVAD explantation. Secondary outcomes included LVAD explant for myocardial recovery, and survival free from stroke (comprising both ischaemic and haemorrhagic stroke), non–central nervous system (non-CNS) bleeding, gastrointestinal bleeding, LVAD-related infections, and device malfunction.

For competing outcome analyses, the following mutually exclusive events were evaluated: death, HT, urgent LVAD explantation, LVAD explantation for myocardial recovery, or ongoing survival on LVAD support at last available follow-up. Follow-up was censored at 5 years from LVAD implant.

As a sensitivity analysis, transplant-free survival was evaluated using the composite end-point of death or HT, whichever occurred first. Patients were censored at LVAD explantation for myocardial recovery, urgent LVAD explantation, or last available follow-up.

### Statistical analysis

Categorical variables were reported as proportions, whereas continuous variables were reported as mean ± SD or median (Q1-Q3), as appropriate. Continuous variables from independent groups were compared by the Student’s *t*-test, or by the Mann-Whitney *U* test and the categorical variables with the chi-square test, as appropriate.

Time-to-event primary analyses were performed using Kaplan-Meier estimates stratified by age classes, with corresponding log-rank tests. Competing-risk analyses were performed using Fine-Gray subdistribution hazard models, considering death, HT, urgent LVAD explantation, and LVAD explantation for myocardial recovery as competing events, and stratified by age group. To complement the Fine-Gray competing-risk analyses, cause-specific Cox proportional hazards models were performed for both death and HT. For each analysis, competing events (HT, LVAD explantation for myocardial recovery, urgent LVAD explantation, or death, as appropriate) were treated as censoring events.

Prespecified subgroup analyses for the primary outcome were performed in patients <50 years according to sex, body mass index (BMI), ethnicity, blood group, device strategy, and INTERMACS profile at implant.

To identify factors associated with successful 5-year LVAD support or LVAD explantation for myocardial recovery among patients <50 years, 2 complementary Cox proportional hazards models were evaluated. A baseline model including only pre-implant variables was developed to identify prognostic factors present at LVAD implantation. In addition, a second model incorporating time-dependent covariates was constructed to explore the association between adverse events occurring during LVAD support and subsequent long-term outcomes. To reduce the risk of overfitting, variable selection among baseline covariates was performed using the least absolute shrinkage and selection operator with 10-fold cross-validation. Time-dependent covariates were subsequently incorporated into the final Cox models. Proportional hazards assumptions were assessed using Schoenfeld scaled and unscaled residuals; when violated, interaction terms between log(time) and the relevant covariate were included. Missing data were imputed prior to multivariable modelling using random forest imputation for categorical variables and predictive mean matching for continuous variables, excluding variables with >20% missingness. Random forest imputation was performed with 100 trees and 5 iterations, and predictive mean matching used *k* = 3 to ensure plausible values. Variables with >20% missingness were excluded from multivariable analyses because imputing variables with extensive missingness may introduce unstable estimates and reduce model reliability, a commonly adopted threshold in observational registry analyses. The proportion of missing data for each candidate variable is reported in **Table S1**. To assess the robustness of the imputed analyses, a complete-case sensitivity analysis of the baseline multivariable model was additionally performed.

In addition, the independent association between age and the primary outcome was evaluated using multivariable Cox proportional hazards regression adjusted for clinically relevant baseline covariates. Age was modelled both as a categorical variable and as a continuous variable using restricted cubic splines to explore potential nonlinear associations.

All analyses were conducted using R version 4.5.0 (R Foundation for Statistical Computing). A 2-sided *P*-value <.05 was considered statistically significant.

### Ethics

The study conformed to the principles outlined in the Declaration of Helsinki. The local ethics committees approved the research for all participating EUROMACS centres prior to study inception, and all patients gave written consent to the participating hospitals to share their anonymized data with EUROMACS, as per EUROMACS policy and operational regulations. Written informed consent was obtained from all participants. The data are presented in EUROMACS in de-identified form, also with blinded centre identifiers to the investigators.

## Results

A total of 2850 patients who underwent primary LVAD implantation with an HM3 were included in the final analysis. A study flow chart of included patients is shown in **Figure 1**. Cumulative follow-up amounted to 6820.4 patient-years. Median age was 59 years (IQR 50-65), and the majority were male (85.8%). Patients were stratified by age at implantation into 4 prespecified groups: <50 years (23.5%), 50-59 years (29.7%), 60-69 years (38.0%), and ≥70 years (8.8%). The baseline characteristics of the study cohort stratified by age groups are presented in **Table 1**. Baseline characteristics differed across groups, so that younger patients had a lower burden of cardiovascular and non-cardiovascular comorbidities, were more likely to have non-ischaemic cardiomyopathy, and not to have an implantable cardioverter-defibrillator (ICD) or an HF duration ≥1 year. Younger patients were more likely to undergo LVAD implant with a bridge to transplant, candidacy or recovery intent, and to undergo LVAD implant in inotrope-dependency (INTERMACS 1-3 profiles) and ECMO. Sex distribution was similar across age strata.

**Figure 1.**
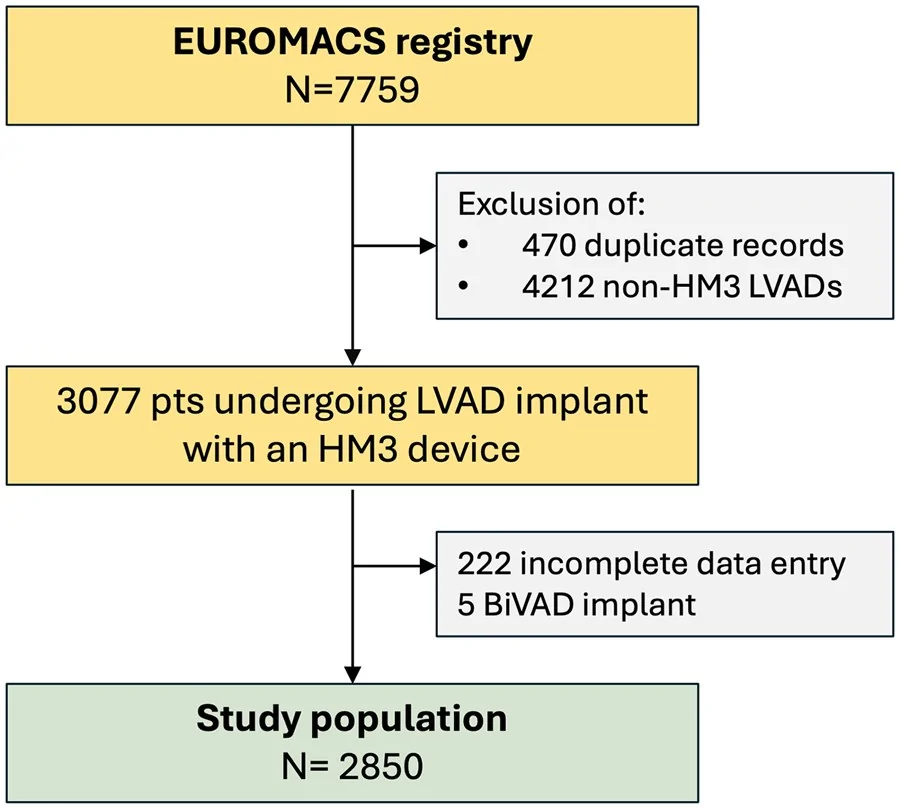
Study Flow Chart. Abbreviations: BiVAD, biventricular assist device; EUROMACS, European Registry for Patients with Mechanical Circulatory Support; HM3, HeartMate 3; LVAD, left ventricular assist device.

**Table 1.** Baseline Characteristics of the Study Cohort Stratified by Age Groups

	Age <50 years *N* = 670	Age 50-59 years *N* = 847	Age 60-69 years *N* = 1083	Age ≥70 years *N* = 250	*P*-value
Age (years), median [IQR]	41 [33-46]	55 [53-58]	64 [62-66]	71 [70-73]	<.001
Male (%)	563 (84.0)	719 (84.9)	940 (86.8)	223 (89.2)	.135
BSA, median (IQR)	2.0 [1.9-2.2]	2.0 [1.9-2.2]	2 [1.9-2.2]	2.0 [1.8-2.1]	<.001
Ethnicity					.168
Caucasian	552 (91.2)	719 (95.0)	871 (94.0)	181 (93.3)	
African American or Black	8 (1.3)	5 (0.7)	6 (0.6)	1 (0.5)	
Other	45 (7.4)	33 (4.4)	50 (5.4)	12 (6.2)	
Blood group (%)					.036
A	258 (38.5)	384 (45.3)	463 (42.8)	106 (42.4)	
AB	48 (7.2)	34 (4.0)	63 (5.8)	7 (2.8)	
B	107 (16.0)	128 (15.1)	149 (13.8)	35 (14.0)	
0	257 (38.4)	301 (35.5)	408 (37.7)	102 (40.8)	
COPD (%)	18 (3.1)	83 (11.1)	140 (14.2)	30 (13.1)	<.001
Peripheral vascular disease (%)	9 (1.5)	39 (5.1)	102 (10.3)	23 (10.6)	<.001
Prior neurological event (%)	49 (8.7)	76 (10.5)	109 (11.4)	28 (12.8)	.248
Active smoking (%)	145 (30.5)	178 (31.6)	152 (22.1)	14 (11.7)	<.001
Diabetes (%)	75 (11.5)	253 (30.9)	373 (34.8)	93 (38.6)	<.001
HF duration ≥1 year (%)	343 (59.4)	557 (74.9)	734 (77.5)	170 (76.6)	<.001
Ischaemic cardiomyopathy (%)	134 (20.0)	405 (47.8)	638 (58.9)	138 (55.2)	<.001
ICD (%)	271 (45.6)	477 (65.6)	645 (71.6)	153 (76.1)	<.001
Device strategy at implant (%)					<.001
Bridge to transplant	311 (46.4)	313 (37.0)	222 (20.5)	11 (4.4)	
Bridge to candidacy	282 (42.1)	368 (43.4)	316 (29.2)	12 (4.8)	
Destination therapy	26 (3.9)	99 (11.7)	492 (45.4)	222 (88.8)	
Bridge to recovery	35 (5.2)	31 (3.7)	21 (1.9)	1 (0.4)	
Cardiac arrest (%)	40 (7.0)	79 (10.7)	84 (8.5)	19 (8.3)	.137
Dialysis (%)	29 (4.6)	42 (5.2)	71 (6.7)	18 (7.4)	.156
Intubation (%)	94 (16.5)	108 (14.7)	128 (13.0)	19 (8.1)	.010
Inotropes (%)	320 (64.0)	375 (61.8)	469 (59.4)	115 (62.8)	.411
IABP (%)	35 (6.2)	66 (9.0)	78 (7.9)	11 (4.7)	.082
pVAD (%)	62 (9.8)	80 (10.0)	89 (8.6)	23 (9.7)	.729
ECMO (%)	87 (13.7)	91 (11.4)	95 (9.2)	18 (7.7)	.012
INTERMACS profile (%)					.007
1	108 (16.5)	124 (15.0)	140 (13.2)	28 (11.6)	
2	164 (25.1)	197 (23.8)	218 (20.6)	40 (16.5)	
3	192 (29.4)	229 (27.7)	341 (32.2)	88 (36.4)	
≥ 4	190 (29.1)	277 (33.5)	360 (34.0)	86 (35.5)	
CPB time (min), median [IQR]	79 [59-106]	84 [61-118]	89 [60-118]	90 [59-118]	.018

Abbreviations: BSA, body surface area; COPD, chronic obstructive pulmonary disease; CPB, cardiopulmonary bypass; ECMO, extracorporeal membrane oxygenation; HF, heart failure; IABP, intra-aortic balloon pump; ICD, implantable cardioverter-defibrillator; INTERMACS, Interagency Registry for Mechanically Assisted Circulatory Support; pVAD, percutaneous ventricular assist device.

### Kaplan-Meier analyses stratified by age

Five-year overall survival differed significantly across age groups, with the highest on-device 5-year survival in patients <50 years and a stepwise decline with increasing age (<50 years: 74.7%; 50-59 years: 65.5%; 60-69 years: 53.9%; ≥70 years: 48.8%; *P* < .001) (**Figure 2**). LVAD explantation for myocardial recovery was also more frequent in younger patients (<50 years: 4.3%; 50-59 years: 1.8%; 60-69 years: 2.3%; ≥70 years: 0.4%; *P* = .031) (**Figure 3**).

**Figure 2.**
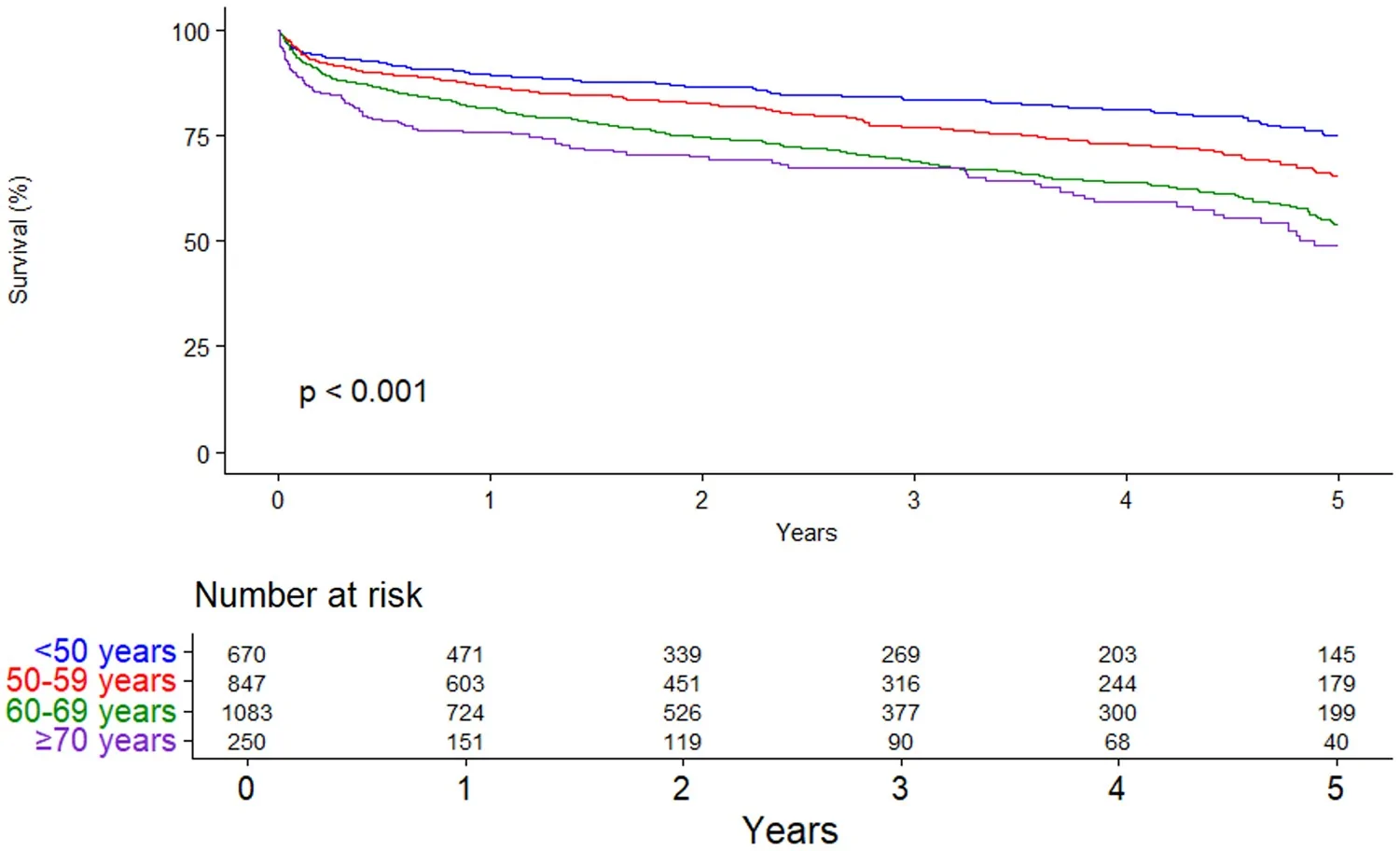
Kaplan-Meier Curves for 5-Year Survival According to Age Groups. Follow-up Censored at HT, LVAD Explantation, or Last Available Visit on LVAD Support. Abbreviations: HT, heart transplantation; LVAD, left ventricular assist device.

**Figure 3.**
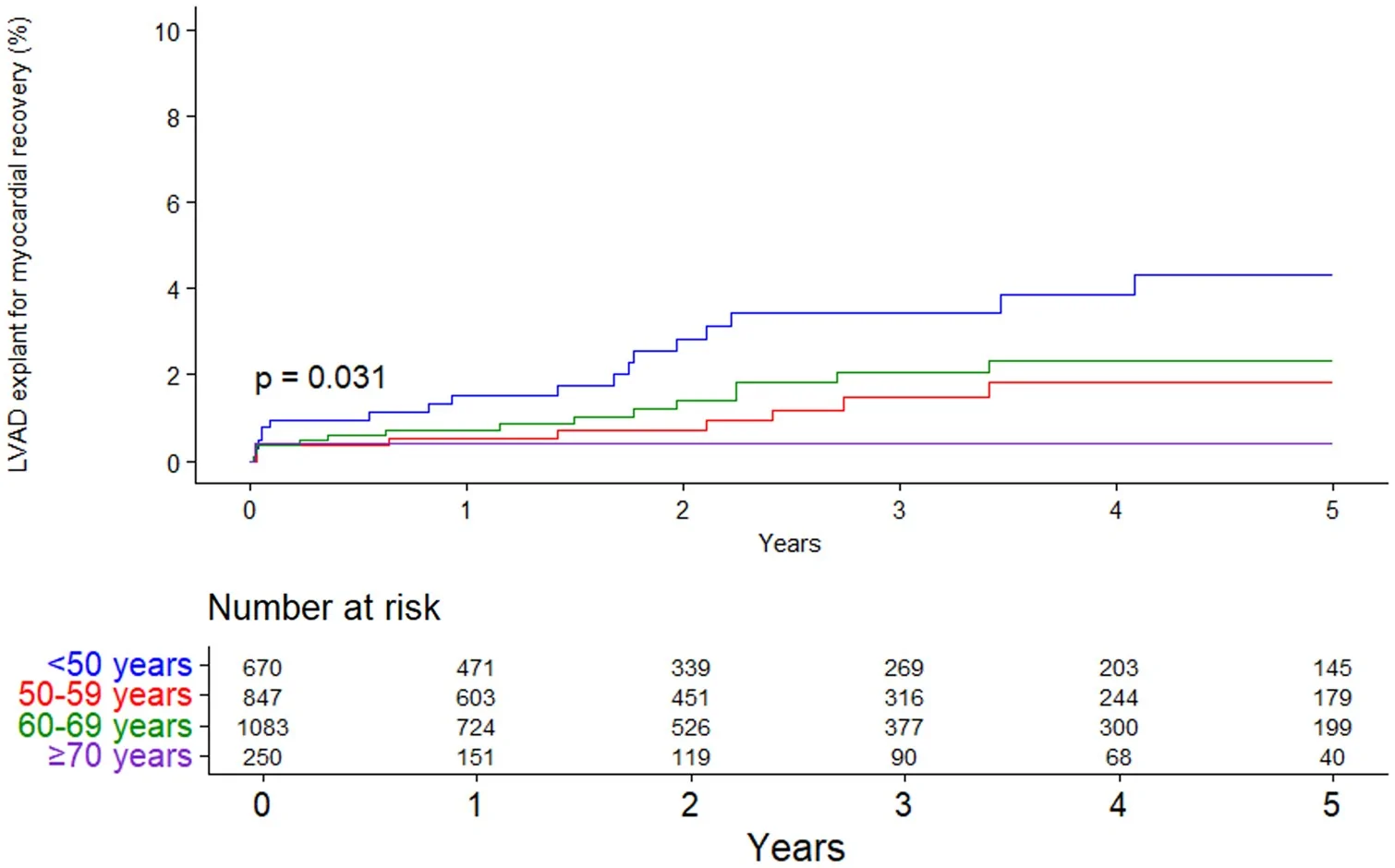
Kaplan-Meier Curves for 5-Year LVAD Explantation for Myocardial Recovery According to Age Groups. Follow-up Censored at HT, Death, or Last Available Visit on LVAD Support. Abbreviations: HT, heart transplantation; LVAD, left ventricular assist device.

Younger patients exhibited significantly higher survival free from non-CNS bleeding (<50 years: 70.2%; 50-59 years: 67.6%; 60-69 years: 63.3%; ≥70 years: 56.9%; *P* < .001) (**Figure 4**), driven by both gastrointestinal (*P* < .001) and non-CNS non-gastrointestinal (*P* = .046) bleedings (**Figures S1 and S2**). Conversely, no differences according to age groups were found in survival free from stroke (<50 years: 89.0%; 50-59 years: 88.4%; 60-69 years: 87.9%; ≥70 years: 86.2%; *P* = .693) with similar data for ischaemic and haemorrhagic strokes (**Figures S3 and S4**), LVAD-related infection (<50 years: 42.3%; 50-59 years: 42.1%; 60-69 years: 47.2%; ≥70 years: 48.2%; *P* = .554) and device malfunction (<50 years: 73.2%; 50-59 years: 72.3%; 60-69 years: 77.2%; ≥70 years: 73.5%; *P* = .492).

**Figure 4.**
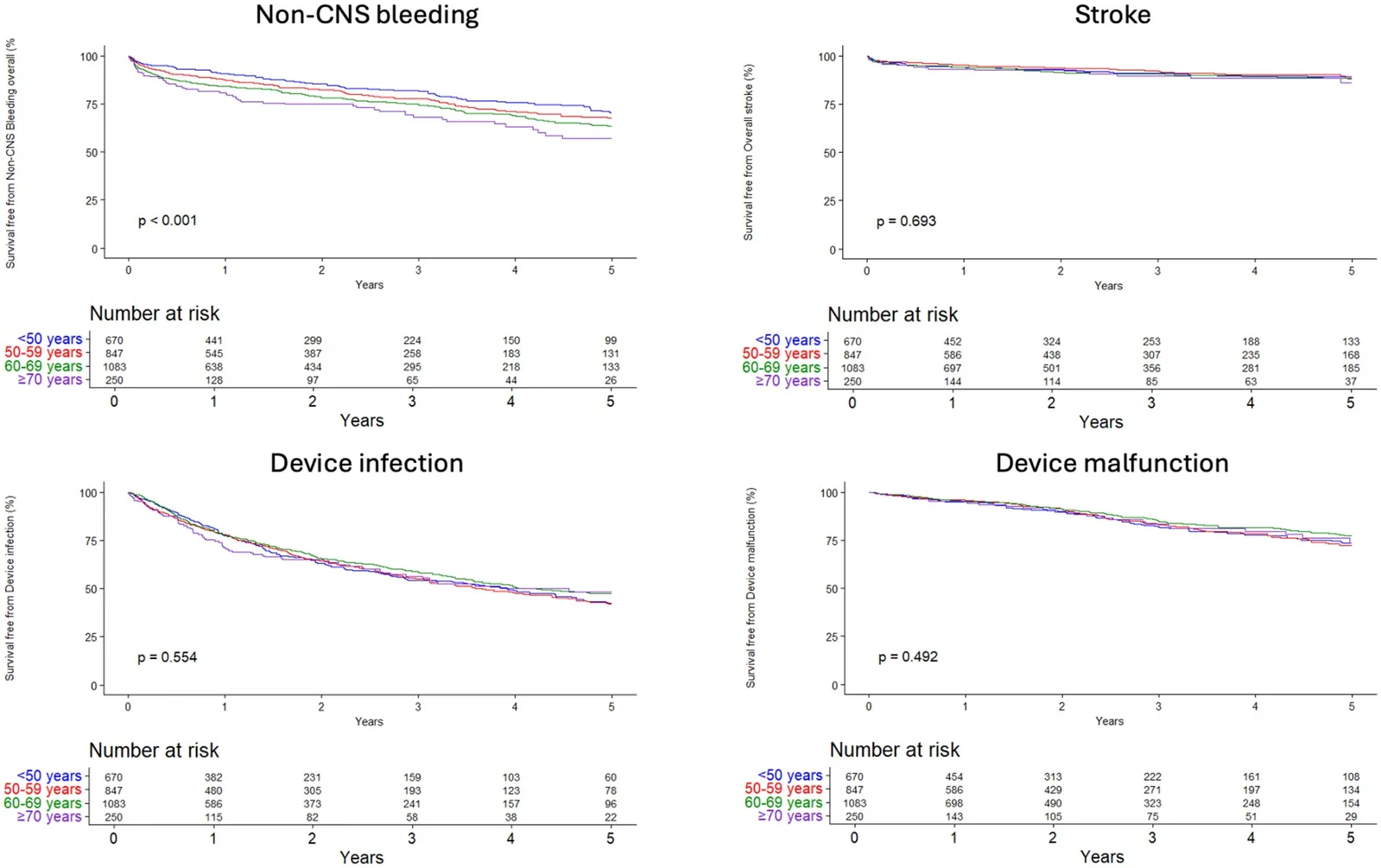
Kaplan-Meier Curves for 5-Year Adverse Events according to Age Groups. Abbreviation: CNS, central nervous system.

As a sensitivity analysis, a composite endpoint of death, stroke, or HT was evaluated (**Table S2**). Compared with the primary on-device survival analysis, age-related differences were attenuated, although patients aged ≥60 years remained at significantly higher risk than those aged <50 years.

As a sensitivity analysis, a 1-year landmark analysis was performed including only patients who remained alive on LVAD support at 1 year (**Figure S6**). While survival differed significantly across age groups during the first year after implantation (log-rank *P* = .003), no significant differences were observed thereafter (log-rank *P* = .454), suggesting that the age-related survival gradient was predominantly driven by events occurring during the early post-implant period.

Finally, to determine whether the observed age gradient remained independent of differences in baseline characteristics, we performed adjusted multivariable Cox regression analyses including major demographic and clinical covariates (**Table S3**). Compared with patients <50 years, increasing age categories remained independently associated with progressively higher mortality risk (50-59 years: adjusted hazard ratio [HR] 1.55, 95% CI, 1.15-2.08; 60-69 years: adjusted HR 1.82, 95% CI, 1.34-2.48; ≥70 years: adjusted HR 2.24, 95% CI, 1.49-3.38; all *P* ≤ .004). When age was analyzed as a continuous variable using restricted cubic splines, mortality risk increased progressively with advancing age, without evidence of a significant nonlinear association (*P* for nonlinearity = .779) (**Figure S7**).

### Subgroup analyses among younger patients (<50 years)

On-device 5-year survival in relevant subgroups of younger patients is presented in **Table 2**. Among younger patients, on-device 5-year survival was comparable regardless of sex, BMI (<25, 25-30, and ≥30), ethnicity (Caucasic vs non-Caucasic), blood group (0 vs others), and device strategy. Conversely, on-device 5-year survival was higher among younger patients undergoing LVAD implant with an outpatient INTERMACS profile (INTERMACS ≥4 vs 1-3: 79.7% vs 74.6%, *P* = .025).

**Table 2.** Five-Year Survival in Relevant Subgroups Among Younger Patients

Subgroups	5-year KM survival on LVAD support	*P*-value
Sex	Female: 73.9% vs male: 74.9%	.353
BMI	<25: 79.6% vs 25-29: 74.8% vs ≥30: 69.2%	.332
Ethnicity	Caucasian vs non-Caucasian: 77.3% vs 57.8%	.172
Blood group	0: 73.2% vs other groups: 75.7%	.435
Device strategy	DT: 55.3% vs other strategies: 75.2%	.966
INTERMACS profile	1-3: 74.6% vs ≥4: 79.7%	.025

Abbreviations: BMI, body mass index; DT, destination therapy; INTERMACS, Interagency Registry for Mechanically Assisted Circulatory Support; KM, Kaplan-Meier.

### Competing outcome analyses

Competing-risk analyses accounting for death, HT, urgent LVAD explantation, and LVAD explantation for myocardial recovery are reported in **Figure 5** (<50 and ≥50 years) and **Figure S5** (for all age groups).

**Figure 5.**
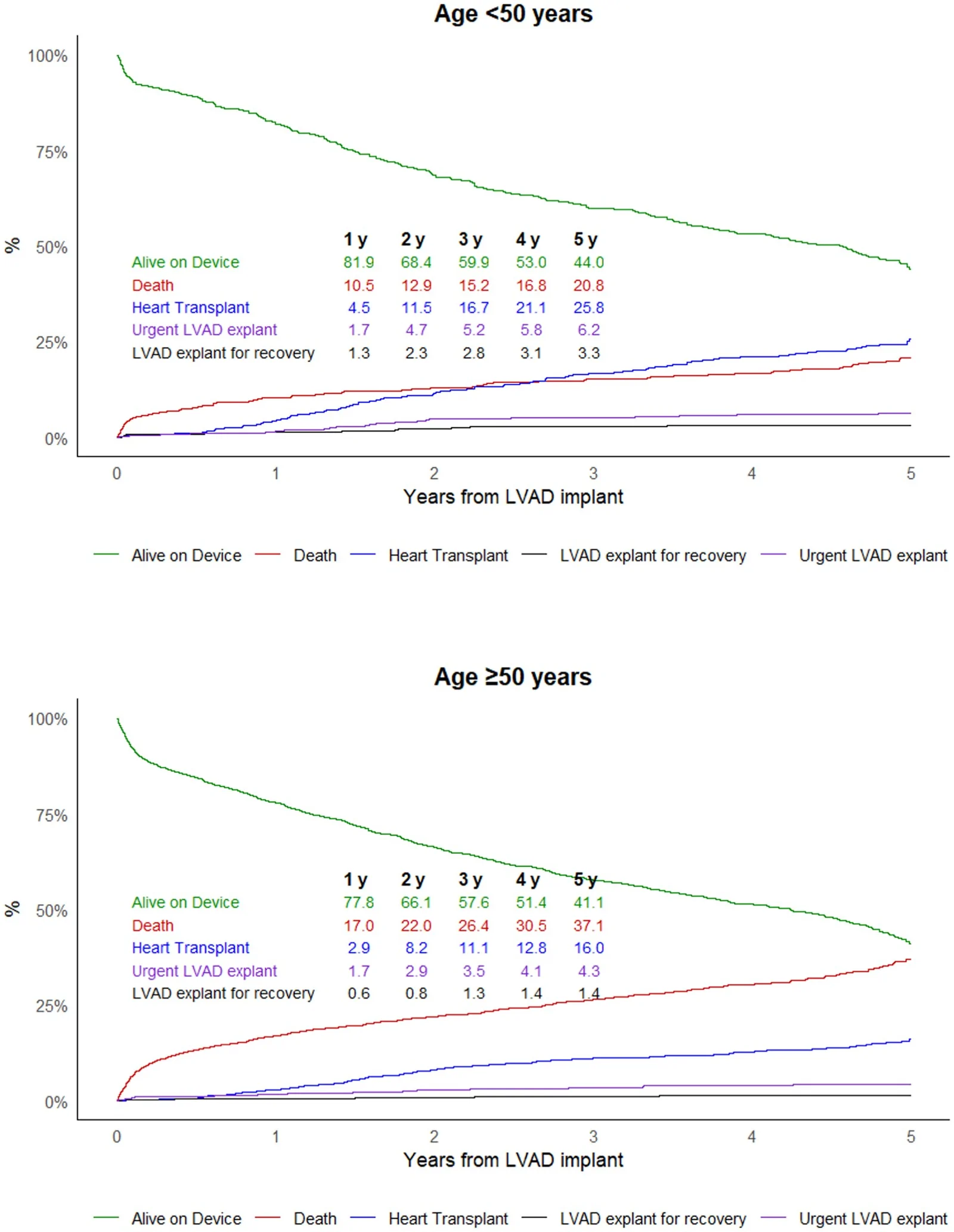
Competitive Outcome Analyses in Younger and Older Patients. Abbreviation: LVAD, left ventricular assist device.

At 5 years, the cumulative incidence of death was lowest among patients <50 years and increased progressively with age (<50 years: 20.8%; 50-59 years: 29.1%; 60-69 years: 40.8%; ≥70 years: 49.5%; *P* < .001). This survival advantage was largely mediated by higher HT rates in younger patients: at 5 years, 25.8% of patients <50 years had undergone HT, compared with 21.0%, 14.1%, and 7.0% in the 50-59, 60-69, and ≥70-year groups, respectively (*P* < .001). Among younger patients, accounting for competitive outcomes, 44.0% were on LVAD support at 5 years. A graphical summary of the distribution of mutually exclusive clinical states at 5 years is provided in **Figure S8**.

As a sensitivity analysis, transplant-free survival, defined as the composite end-point of death or HT, was evaluated according to age groups (**Figure S9**). No significant differences were observed among age groups (log-rank *P* = .210), reflecting the higher rate of HT in younger patients.

To further characterize the relationship between age and competing events, cause-specific Cox regression analyses were performed. Compared with patients <50 years, increasing age was independently associated with a progressively higher cause-specific hazard of death (50-59 years: HR 1.40, 95% CI, 1.11-1.77; 60-69 years: HR 2.03, 95% CI, 1.64-2.51; ≥70 years: HR 2.49, 95% CI 1.89-3.27; all *P* ≤ 0.004). Conversely, the cause-specific hazard of HT progressively decreased with increasing age (50-59 years: HR 0.84, 95% CI, 0.65-1.07; 60-69 years: HR 0.60, 95% CI, 0.47-0.78; ≥70 years: HR 0.22, 95% CI, 0.11-0.43) (**Table S4**).

### Independent predictors of survival on LVAD support or explant for recovery among younger patients

In multivariable Cox regression analyses, several factors were independently associated with the composite outcome of survival on LVAD support or LVAD explantation for myocardial recovery at 5 years in younger patients (**Table 3**). Favourable predictors included INTERMACS ≥4 at LVAD implant (adj-HR 0.47; 95% CI, 0.29-0.77; *P* = .002), no ICD (adj-HR 0.67; 95% CI, 0.53-0.87; *P* = .002), no pre-LVAD tMCS (adj-HR 0.62; 95% CI, 0.40-0.97; *P* = .035), no bleeding during LVAD support (adj-HR 0.62; 95% CI, 0.43-0.90; *P* = .011), and no stroke during LVAD support (adj-HR 0.59; 95% CI, 0.37-0.92; *P* = .020). The baseline-only multivariable model yielded highly consistent results, confirming INTERMACS profile ≥4, absence of pre-LVAD temporary mechanical circulatory support, and absence of ICD as the principal baseline factors associated with successful long-term LVAD support (**Table S5**).

**Table 3.** Multivariable Analysis of Predictors of the Composite Outcome of Survival on LVAD Support or LVAD Explantation for Myocardial Recovery at 5 Years in Patients <50 Years

Variable	HR	95% CI		*P*-value
ICD	1.48	1.16	1.89	**.002**
INTERMACS ≥4	0.47	0.29	0.77	**.002**
Pre-LVAD tMCS	1.60	1.03	2.48	**.035**
Cardiac Arrest	1.24	0.79	1.95	.35
Dialysis	1.31	0.72	2.36	.38
Bleeding on LVAD support	1.60	1.11	2.30	**.011**
Stroke on LVAD support	1.70	1.09	2.67	**.020**
Device malfunction on LVAD support	0.85	0.50	1.43	.528
Device infection on LVAD support	1.02	0.75	1.38	.924

HRs and 95% CIs are derived from a Cox proportional hazards model. Baseline covariates were prespecified as described in the Methods and selected using LASSO with 10-fold cross-validation to reduce overfitting. Time-dependent covariates included adverse events occurring during LVAD support (bleeding, stroke, device malfunction, and device-related infection). Variables retained in the final multivariable model are shown in bold.

Abbreviations: HR, hazard ratio; ICD, implantable cardioverter-defibrillator; INTERMACS, Interagency Registry for Mechanically Assisted Circulatory Support; LVAD, left ventricular assist device; tMCS, temporary mechanical circulatory support.

The complete-case sensitivity analysis yielded results broadly consistent with the primary imputed model (**Table S6**).

## Discussion

This EUROMACS analysis, broadly representing the contemporary European real-world cohort of LVAD patients, provides several key insights on the durability of LVAD support in younger patients that may inform management strategies for heart replacement therapies at the individual patient and system levels.

First, younger patients experienced significantly better 5-year overall survival compared with older recipients, accompanied by lower rates of hemocompatibility-related adverse events and a higher likelihood of LVAD explantation for myocardial recovery. These findings confirm a strong association between age and the long-term benefit from contemporary LVAD therapy. This association remained significant after adjustment for major baseline confounders and was confirmed when age was modelled as a continuous variable, supporting an independent and approximately linear relationship between advancing age and long-term mortality risk. Although residual confounding cannot be completely excluded, age should not be viewed simply as another baseline covariate. Rather, it represents a clinically meaningful surrogate integrating biological reserve, physiological resilience, and comorbidity burden, all of which influence tolerance to advanced HRTs. Consequently, while treatment decisions at the individual patient level should always rely on a comprehensive multiparametric assessment, age remains a simple and clinically useful marker for population-level risk stratification. Interestingly, landmark analysis demonstrated that the age-related survival advantage was largely confined to the first year after LVAD implantation, whereas long-term survival among patients successfully traversing this early period was comparable across age groups. This finding suggests that age primarily influences early vulnerability following LVAD implantation rather than subsequent long-term device support. Second, the overall 5-year survival of 74.7% among patients <50 years was in the range of that reported for HT, highlighting the favourable mid-term outcomes achievable with contemporary LVAD support in carefully selected younger patients. Third, competitive outcome analyses demonstrated that this survival advantage was at least partly mediated by higher HT rates in younger patients, such that 25.8% had undergone transplantation by 5 years. Fourth, in the time-dependent model, the occurrence of major hemocompatibility-related adverse events during LVAD support was associated with reduced long-term survival. These findings should be interpreted as mechanistic associations occurring during follow-up rather than baseline prognostic factors for patient selection, as confirmed by the separate baseline multivariable model. The apparent protective association of absence of ICD and pre-implant temporary mechanical circulatory support likely reflects residual confounding by baseline disease severity rather than a causal effect.

Taken together, these findings refine prior registry evidence and help further shape the discussion of a net prolongation of life paradigm among younger patients undergoing LVAD implantation, providing contemporary European data that support further evaluation of sequential heart replacement strategies. Our data highlight the optimal mid-term outcomes of LVAD support in younger patients^5^^,^^6^ and, importantly, extend prior observations to a large, contemporary European population. This generalizability is non-trivial, as European cohorts differ from North American populations with respect to baseline characteristics, ethnic composition, healthcare systems, transplant allocation policies, waiting list, and LVAD indications and management strategies.^12^

In this setting, we observed a 5-year survival of 74.7% in patients <50 years, closely aligned with outcomes reported in recent US registry analyses (72.6%).^5^ This concordance suggests that the prognostic advantage associated with younger age is robust across divergent healthcare systems and reinforces the external validity of prior INTERMACS findings. Similarly, patterns of adverse events during LVAD support were largely consistent with prior INTERMACS reports.^5^ Younger patients experienced a significantly lower incidence of hemocompatibility-related adverse events, driven primarily by a reduction in non-CNS haemorrhagic complications, whereas ischaemic events remained comparatively infrequent across all age strata. In contrast, LVAD-related infections were not meaningfully modulated by age, and infection-free survival remained disappointingly low across all age groups. These findings identify infection as a persistent, age-independent limitation of long-term LVAD support and a major obstacle to further extending durable support, even among younger patients. ^5^^,^^13^^,^^14^ This finding underscores infections as a major obstacle to the pump-patient continuum and a critical barrier to fully realizing the net prolongation of life concept, even in younger and otherwise favourable candidates. Future strategies including more flexible, antimicrobial-coated drivelines or fully implantable systems may address this persistent limitation.

In line with prior evidence, we observed a higher rate of LVAD explantation for myocardial recovery in younger patients, reaching 4.3% at 5 years.^15–18^ To our knowledge, this is the first report describing recovery rates at this time horizon in a large, unselected European registry reflecting contemporary practice. This finding suggests an additional potential benefit of prolonged LVAD support in selected younger patients, although its implications for the optimal sequencing of heart replacement therapies remain to be established. As articulated in recent conceptual works, durable LVAD support may provide sufficient ventricular unloading to enable myocardial recovery in a subset of patients, allowing prolonged periods free from both heart failure and mechanical support.^16^^,^^17^^,^^19^^,^^20^ Importantly, even when recovery is not durable and relapse occurs, patients who subsequently undergo HT appear to have post-transplant outcomes comparable to unselected transplant cohorts, suggesting that recovery-first trajectories do not compromise later transplant success.^19^ Although recovery remained uncommon, its higher incidence among younger patients provides an additional biological rationale for further investigation of sequential heart replacement strategies. Moreover, even when formal explantation rates remain low, myocardial recovery may be underestimated in contemporary practice. Partial or near-complete recovery has been described in a larger proportion of LVAD-supported patients than those ultimately undergoing explantation, reflecting both clinical caution and limited willingness to pursue device removal in the absence of standardized recovery pathways. More systematic implementation of optimized guideline-directed medical therapy during LVAD support,^21^ together with structured mechanical unloading and recovery protocols, may increase the proportion of patients achieving meaningful myocardial recovery.^15^^,^^22–24^ On this basis, the number of patients who may derive benefit from an LVAD-first approach could expand beyond those currently identified by explantation rates alone.

A novel and clinically relevant finding of the present analysis is that the survival benefit observed in younger patients was at least partly mediated by higher utilization of HT. This observation helps refine the contemporary value of an LVAD-first paradigm beyond what has been previously inferred from INTERMACS and other analyses.^5^^,^^6^ Indirectly consistent findings have been reported in analyses comparing LVAD-first vs transplant-first strategies, where HT rates of 23.6% at 2 years contributed to similar outcomes between strategies.^6^ Together, these findings suggest that prolonged LVAD support does not necessarily preclude timely access to HT when clinically indicated, although this hypothesis requires prospective validation. We did not analyse the specific indications for HT, and it therefore remains unclear whether these transplants were performed under urgent or elective circumstances, particularly since registry data may not fully distinguish patients with progressive LVAD-related complications who are not “crashing” and are therefore transplanted under formally elective conditions. Future analyses will be required to clarify this important distinction and further delineate optimal timing and selection criteria for transplantation following LVAD implantation.

Within the younger population, our findings suggest that the potential benefit of an LVAD-first strategy may be particularly relevant in specific subgroups. Patients implanted in INTERMACS profile ≥4 appeared to derive the greatest mid-term benefit from durable LVAD support, consistent with prior data from the INTERMACS registry.^25^ This observation suggests that some selected patients with lower acuity profiles might benefit from earlier referral and implantation before advanced end-organ dysfunction develops.^26^^,^^27^ The TEAM-HF trial (NCT06526195) will help to further clarify this clinical hypothesis.

Importantly, the durability of LVAD support—and thus the magnitude of net survival gain—remains closely linked to the burden of device-related complications. In the present analysis, hemocompatibility-related adverse events, driven by bleedings, were independently associated with reduced on-device survival. Encouragingly, hemocompatibility management continues to evolve. Aspirin-sparing strategies, lower-intensity anticoagulation protocols, and exploration of direct oral anticoagulants are underway and may further rebalance the residual bleeding-ischaemic trade-off in contemporary devices.^3^^,^^4^^,^^28^ As these strategies mature and are validated in randomized trials, they may further enhance the survival and quality-of-life profile of LVAD therapy, particularly in younger candidates with longer anticipated duration of support.

Beyond hemocompatibility, haemodynamic-related events represent another key determinant of LVAD durability. Persistent aortic insufficiency, right ventricular failure, and subclinical congestion may progressively erode the long-term benefit of LVAD therapy.^21^^,^^29^ Emerging strategies, including remote haemodynamic monitoring^30^ and transcatheter correction of aortic insufficiency with tailored devices,^31^ may mitigate this limitation and extend durable support duration. In parallel, ongoing technological evolution—including device miniaturization, development of fully implantable or wireless energy transmission systems, and more refined physiologic flow modulation—has the potential to enhance both the safety and tolerability of long-term LVAD support. As complication rates decline and the day-to-day burden of device therapy diminishes, the relative disadvantages historically associated with durable mechanical support may become less pronounced. Such progress may progressively reshape how clinicians and patients weigh LVAD therapy against transplantation and prompt reconsideration of traditional thresholds for timing and candidacy within the heart replacement spectrum.

Finally, while our findings strengthen the biological and clinical plausibility of an LVAD-first strategy in selected younger patients, they must be interpreted within a broader patient-centred framework. Contemporary LVAD therapy remains associated with meaningful lifestyle implications, including driveline care, external batteries, limitations in physical autonomy, device-related anxiety, and psychosocial impact. For many patients, these quality-of-life considerations may weigh heavily and may not be perceived as adequately balanced by a potential prognostic advantage. Quality-of-life assessments and patient-reported outcomes should be integral to future comparative effectiveness research.

Accordingly, these data should not be interpreted as advocating a one-size-fits-all paradigm shift towards universal LVAD-first implantation in younger transplant-eligible patients. Rather, age should be considered as 1 component of a comprehensive clinical assessment integrating comorbidity burden, disease severity, transplant candidacy, and patient preferences, in keeping with contemporary EACTS recommendations on durable mechanical circulatory support, which emphasize individualized multidisciplinary patient selection rather than therapeutic intent alone.^32^ Similarly, the recent EACTS/STS/AATS guidance frames mechanical circulatory support as part of an integrated heart replacement pathway, in which patients may transition between temporary support, durable LVAD therapy, myocardial recovery, and HT according to their evolving clinical status.^33^ Our findings should therefore be interpreted within this contemporary conceptual framework, while recognizing that the absence of post-transplant follow-up precludes any conclusions regarding the optimal sequencing of heart replacement therapies.

Moreover, they should inform a nuanced and balanced discussion between physicians and patients regarding the potential trade-offs between sequential heart replacement strategies. Decisions should integrate not only survival probabilities but also patient values, life goals, tolerance for device-related burden, and individual risk profiles. A truly modern application of the net prolongation of life concept must therefore remain rooted in shared decision-making and personalized medicine.

### Limitations

Several limitations should be acknowledged. First, the inherent constraints of large, real-world registries apply, including residual confounding, missing data, and lack of centralized adjudication for all outcomes. Second, post-HT outcomes of LVAD patients undergoing HT were not captured in the registry. This is currently the most important limitation, shared with other large datasets, precluding a complete assessment of the long-term trade-offs of an LVAD-first strategy. While some studies suggest no adverse impact of LVAD status on post-transplant outcomes, others indicate a potential association between duration of LVAD support and post-transplant prognosis. Accordingly, our findings should be interpreted as describing age-specific outcomes during LVAD support rather than supporting 1 heart replacement strategy over another. Further dedicated analyses will be needed to clarify this issue and better define the long-term implications of sequential heart replacement strategies. Of note, to more accurately gauge HT eligibility, a composite end-point of mortality plus debilitating stroke—events that preclude or substantially diminish sequential HT feasibility—could enhance future analyses. Third, quality-of-life data were not systematically captured, precluding assessment of patient-reported outcomes. Fourth, the present analysis could not account for centre- or healthcare system-level heterogeneity. Differences in institutional experience, transplant activity, organ allocation policies, anticoagulation protocols, and myocardial recovery practices may have influenced both treatment pathways and long-term outcomes. Consequently, part of the observed age-related differences may reflect institutional or regional practice variation rather than biological differences alone. Future analyses incorporating centre-level effects may help further refine these findings.

## Conclusions

In a large contemporary European cohort, younger patients undergoing durable LVAD implantation demonstrated favourable 5-year outcomes, comparable to HT, with lower bleeding complications and higher rates of myocardial recovery. Accounting for competing outcomes, 44% remained on LVAD support at 5 years, while 26% underwent HT. These findings support further evaluation of LVAD-first strategies in selected younger patients to potentially maximize lifetime survival.

## Supplementary Material

ezag234_Supplementary_Data

## Data Availability

The data that support the findings of this study were obtained from participating centres and are subject to institutional and regulatory restrictions. De-identified data may be shared upon reasonable request to the corresponding author, pending approval by the study steering committee and in compliance with data protection regulations.
