# Supplementary material for: Long-Term Outcomes of Contemporary Durable Left Ventricular Assist Device Support in Younger Patients: A EUROMACS Analysis
Source: Eur J Cardiothorac Surg. 2026 Oct 7;68(11-12):ezag234. doi: 10.1093/ejcts/ezag234 (PMC13735509; doi:10.1093/ejcts/ezag234)
Supplement: Supplementary file 1 [file ezag234_supplementary_data.zip › Supplementary Appendix.docx]

**Supplementary Appendix**

**Supplementary Table 1. Proportion of missing data for candidate baseline variables considered for multivariable analyses.**

| Variable | % Missing | Variable | % Missing |
| --- | --- | --- | --- |
| ModifierFF | 40.4 | Intubation | 11.3 |
| ModifierTCSTemporaryCirculator | 39.5 | COPD | 10.6 |
| HistoryOfPreviousAlcoholAbuse | 38.9 | PVD | 9.0 |
| ModifierAArrhythmia | 37.7 | ECMO | 5.4 |
| DrugAbuse | 37.1 | OtherVAD | 5.2 |
| smoking | 35.2 | Dialysis | 3.6 |
| Maritalstatus | 27.6 | INTERMACS_Profile | 2.4 |
| IVInotropeTherapyImmediatelyPr | 27.1 | Diabetes | 2.3 |
| CPBTime | 15.2 | BSA | 1.2 |
| CurrentICDDeviceInPlace | 15.0 | AgeInYears | 0.0 |
| HistoryOfNeurologicalEvent | 13.5 | Gender | 0.0 |
| Race | 12.9 | Bloodtype | 0.0 |
| HF_history | 12.6 | cardiomyopathy | 0.0 |
| IABP | 11.9 | CurrentDeviceStrategy | 0.0 |
| CardiacArrest | 11.4 |  |  |

Percentage of missing observations for all candidate baseline variables evaluated for inclusion in the multivariable Cox regression analyses. Variables with >20% missing data were excluded from multivariable modelling before multiple imputation, as prespecified in the statistical analysis plan.

**Supplementary Table 2. Sensitivity analysis of the primary endpoint using a composite of death, stroke, or heart transplantation according to age groups.**

|  | HR | 95% CI | | p |
| --- | --- | --- | --- | --- |
| Age <50 | REF. |  |  |  |
| Age 50-59 | 1.075 | 0.916 | 1.262 | 0.373 |
| Age 60-69 | 1.274 | 1.097 | 1.479 | 0.002 |
| Age ≥70 | 1.278 | 1.023 | 1.596 | 0.030 |

Multivariable Cox proportional hazards analysis evaluating the composite endpoint of all-cause death, stroke, or heart transplantation according to age categories. Hazard ratios (HRs) are reported using patients aged <50 years as the reference group.

**Supplementary Table 3. Multivariable Cox regression analyses evaluating the independent association between age and 5-year on-device survival.**

|  | HR | 95% CI |  | p |
| --- | --- | --- | --- | --- |
| Age < 50 | REF. |  |  |  |
| Age 50-59 | 1.545 | 1.146 | 2.084 | 0.004 |
| Age 60-69 | 1.824 | 1.341 | 2.481 | <0.001 |
| Age ≥70 | 2.241 | 1.487 | 3.378 | <0.001 |
| Male | 1.300 | 0.958 | 1.763 | 0.092 |
| Diabetes | 1.158 | 0.949 | 1.413 | 0.149 |
| COPD | 1.124 | 0.851 | 1.485 | 0.410 |
| PAD | 1.252 | 0.894 | 1.753 | 0.191 |
| Isc cardiomyopathy | 1.097 | 0.901 | 1.337 | 0.357 |
| INTERMACS ≥4 | 0.779 | 0.634 | 0.958 | 0.018 |
| Device strategy | 1.202 | 0.955 | 1.512 | 0.117 |
| ECMO | 2.183 | 1.664 | 2.863 | <0.001 |
| ICD | 0.965 | 0.766 | 1.216 | 0.761 |
| HF history | 0.936 | 0.739 | 1.188 | 0.588 |

Adjusted Cox proportional hazards models evaluating the association between age and the primary endpoint after adjustment for clinically relevant baseline covariates (sex, diabetes mellitus, COPD, peripheral vascular disease, ischemic cardiomyopathy, INTERMACS profile, device strategy, ECMO at implant, ICD, and heart failure duration).

**Supplementary Table 4. Cause-specific Cox regression analyses for death and heart transplantation according to age groups.**

| Death | | | | |
| --- | --- | --- | --- | --- |
|  | HR | 95% CI | | p |
| Age <50 | REF. |  |  |  |
| Age 50-59 | 1.402 | 1.112 | 1.768 | 0.004 |
| Age 60-69 | 2.026 | 1.637 | 2.507 | <0.001 |
| Age ≥70 | 2.485 | 1.889 | 3.270 | <0.001 |
| HT | | | | |
|  | HR | 95% CI | | p |
| Age <50 | REF. |  |  |  |
| Age 50-59 | 0.836 | 0.654 | 1.069 | 0.152 |
| Age 60-69 | 0.604 | 0.468 | 0.780 | <0.001 |
| Age ≥70 | 0.220 | 0.112 | 0.433 | <0.001 |

Cause-specific Cox proportional hazards models evaluating the independent association between age groups and (A) all-cause death and (B) heart transplantation. For each analysis, competing events were treated as censoring events, complementing the Fine–Gray competing-risk analyses presented in the main manuscript.

**Supplementary Table 5. Baseline multivariable Cox model identifying predictors of successful long-term LVAD support among patients aged <50 years.**

|  | HR | 95% CI | | p |  |
| --- | --- | --- | --- | --- | --- |
| ICD | 1.48 | 1.16 | 1.88 | 0.002 | Fixed |
| INTERMACS ≥4 | 0.47 | 0.29 | 0.77 | 0.002 | Fixed |
| tMCS prior to LVAD | 1.58 | 1.02 | 2.44 | 0.042 | Fixed |
| Cardiac Arrest | 1.25 | 0.79 | 1.96 | 0.339 | Fixed |
| Dialysis | 1.46 | 0.82 | 2.60 | 0.195 | Fixed |

Baseline prognostic model including only pre-implant variables associated with the composite endpoint of survival on LVAD support or LVAD explantation for myocardial recovery at 5 years. This analysis was performed separately from the time-dependent model to distinguish baseline prognostic factors from events occurring during follow-up.

**Supplementary Table 6. Complete-case sensitivity analysis of the baseline multivariable Cox regression model in patients aged <50 years.**

|  | HR | 95% CI | | p |
| --- | --- | --- | --- | --- |
| **ICD** | **1.99** | **1.45** | **2.72** | **0.000** |
| **INTERMACS≥4** | **0.64** | **0.46** | **0.90** | **0.010** |
| CPB time | 1.00 | 1.00 | 1.01 | 0.270 |
| Race | 0.59 | 0.34 | 1.00 | 0.052 |
| Diabetes | 0.84 | 0.51 | 1.39 | 0.501 |
| Male gender | 1.19 | 0.75 | 1.88 | 0.463 |
| Ischemic Cardiomyopathy | 1.84 | 1.26 | 2.67 | 0.001 |

Sensitivity analysis including only patients with complete baseline data.

**Supplementary Figure 1. Kaplan Meier curves for 5 year gastrointestinal bleeding according to age groups.**

Cumulative incidence curves for gastrointestinal bleeding according to age groups, accounting for the competing risk of death, heart transplantation, LVAD explantation for myocardial recovery, and urgent LVAD explantation using Fine–Gray competing-risk analysis.

**Supplementary Figure 2. Kaplan Meier curves for 5 year non-CNS non-gastrointestinal bleeding according to age groups.**

Cumulative incidence curves for non-gastrointestinal bleeding according to age groups, accounting for the competing risk of death, heart transplantation, LVAD explantation for myocardial recovery, and urgent LVAD explantation using Fine–Gray competing-risk analysis.

**Supplementary Figure 3. Kaplan Meier curves for 5 year ischemic stroke according to age groups.**

Cumulative incidence curves for ischemic stroke according to age groups, accounting for the competing risk of death, heart transplantation, LVAD explantation for myocardial recovery, and urgent LVAD explantation using Fine–Gray competing-risk analysis.

**Supplementary Figure 4. Kaplan Meier curves for 5 year hemorrhagic stroke according to age groups.**

Cumulative incidence curves for hemorrhagic stroke according to age groups, accounting for the competing risk of death, heart transplantation, LVAD explantation for myocardial recovery, and urgent LVAD explantation using Fine–Gray competing-risk analysis.

**Supplementary Figure 5. Competitive outcome analyses according to age strata.**

Competing-outcome analysis showing the cumulative incidence of all-cause death, heart transplantation, LVAD explantation for myocardial recovery, and urgent LVAD explantation according to the four predefined age groups. Cumulative incidence functions were estimated using Fine–Gray competing-risk models.

**Supplementary Figure 6. One-year landmark survival analysis according to age groups.**

Kaplan–Meier survival curves showing **(A)** overall survival during the first year following LVAD implantation and **(B)**conditional survival among patients who remained alive and on LVAD support at 1 year. Survival was compared across the four predefined age groups using the log-rank test.

**Supplementary Figure 7. Adjusted association between age and 5-year mortality using restricted cubic splines.**

Adjusted hazard ratio for the primary endpoint according to age modelled as a continuous variable using restricted cubic splines within a multivariable Cox proportional hazards model. The model was adjusted for sex, diabetes mellitus, chronic obstructive pulmonary disease, peripheral vascular disease, ischemic cardiomyopathy, INTERMACS profile, device strategy, extracorporeal membrane oxygenation at implantation, implantable cardioverter-defibrillator, and heart failure duration. The solid line represents the adjusted hazard ratio, and the shaded area represents the 95% confidence interval.

**Supplementary Figure 8. Five-year clinical status according to age group.**

Distribution of mutually exclusive clinical states at 5 years after LVAD implantation in patients aged **<50 years** and **≥50 years**. Clinical states include ongoing LVAD support, heart transplantation, LVAD explantation for myocardial recovery, urgent LVAD explantation, and death.

**Supplementary Figure 9. Transplant-free survival according to age groups.**

Kaplan–Meier curves for transplant-free survival, defined as freedom from the composite endpoint of all-cause death or heart transplantation, according to the four predefined age groups. Patients were censored at LVAD explantation for myocardial recovery, urgent LVAD explantation, or last available follow-up.
